# Behavioral Nudging With Generative AI for Content Development in SMS Health Care Interventions: Case Study

**DOI:** 10.2196/52974

**Published:** 2024-10-15

**Authors:** Rachel M Harrison, Ekaterina Lapteva, Anton Bibin

**Affiliations:** 1 GenAI Lab Ophiuchus LLC Dover, DE United States; 2 Institute of Psychology Russian Academy of Sciences Moscow Russian Federation; 3 Skoltech AI (Centers for Research, Education, and Innovation) Skolkovo Institute of Science and Technology Moscow Russian Federation

**Keywords:** generative artificial intelligence, generative AI, prompt engineering, large language models, GPT, content design, brief message interventions, mHealth, behavior change techniques, medication adherence, type 2 diabetes

## Abstract

**Background:**

Brief message interventions have demonstrated immense promise in health care, yet the development of these messages has suffered from a dearth of transparency and a scarcity of publicly accessible data sets. Moreover, the researcher-driven content creation process has raised resource allocation issues, necessitating a more efficient and transparent approach to content development.

**Objective:**

This research sets out to address the challenges of content development for SMS interventions by showcasing the use of generative artificial intelligence (AI) as a tool for content creation, transparently explaining the prompt design and content generation process, and providing the largest publicly available data set of brief messages and source code for future replication of our process.

**Methods:**

Leveraging the pretrained large language model GPT-3.5 (OpenAI), we generate a collection of messages in the context of medication adherence for individuals with type 2 diabetes using evidence-derived behavior change techniques identified in a prior systematic review. We create an attributed prompt designed to adhere to content (readability and tone) and SMS (character count and encoder type) standards while encouraging message variability to reflect differences in behavior change techniques.

**Results:**

We deliver the most extensive repository of brief messages for a singular health care intervention and the first library of messages crafted with generative AI. In total, our method yields a data set comprising 1150 messages, with 89.91% (n=1034) meeting character length requirements and 80.7% (n=928) meeting readability requirements. Furthermore, our analysis reveals that all messages exhibit diversity comparable to an existing publicly available data set created under the same theoretical framework for a similar setting.

**Conclusions:**

This research provides a novel approach to content creation for health care interventions using state-of-the-art generative AI tools. Future research is needed to assess the generated content for ethical, safety, and research standards, as well as to determine whether the intervention is successful in improving the target behaviors.

## Introduction

### Overview

Health care interventions involving written communication play a pivotal role in disseminating critical information to patients and promoting positive health outcomes. However, the process of crafting effective health care content has historically been labor-intensive, time-consuming, and often lacks the necessary uniformity and transparency required for rigorous research and development.

We propose the application of generative artificial intelligence (AI) technologies to address the pressing need for efficient and transparent content creation in health care interventions. In particular, we focus on harnessing the capabilities of pretrained large language models (LLMs), which are sophisticated AI systems designed to understand and generate human-like text (refer to subsection Generative AI With LLMs). By using these rapidly growing technologies, we aim to assist researchers in the content creation process, making it more accessible, systematic, and adaptable. As a tangible example, we introduce the first publicly available data set of AI-generated brief messages tailored for individuals with type 2 diabetes, specifically targeting medication adherence, a critical aspect of diabetes management. Notably, our data set of 1150 messages also stands as the current largest data set of health care intervention messages publicly available. Furthermore, we make our source code replicable and accessible to the research community while providing a comprehensive breakdown of our design process. In doing so, we seek to use generative AI to pave the way for a new era of health care intervention content development, one characterized by transparency, efficiency, and scientific rigor. Our main contributions are as follows:

1. Present a generative AI approach to content creation in brief message health care interventions
2. Illustrate the process of prompt engineering for content design within a particular theoretical framework
3. Provide the first publicly available data set of AI-generated intervention messages and release the source code as a resource for future research.

### Mobile Health Interventions

In the ever-growing landscape of health care, effective communication is essential to enhancing preventive measures and developing intervention strategies that improve public health outcomes. Each year, a great number of new intervention studies are added to the health care literature [1,2]. However, with the growth in the quantity of interventions often comes an increase in their technical complexity, especially in the area of mobile health (mHealth). Many of these interventions are delivered through proprietary apps or other nonstandardized platforms, which complicates their integration into future programs and often causes their results to be obfuscated by the unique specifics of their deployment. This not only makes them challenging to apply elsewhere but also ensures that their development is both time-consuming and resource-intensive [3]. In addition, while research into mHealth has boomed in the last decade, studies suggest that the overall success rate of most mHealth interventions is not exactly clear, despite the strong interest and obvious potential such interventions have [4,5]. Ensuring that these interventions are feasible, effective, and sustainable, is vital for preventing unnecessary research waste.

Within the sphere of mHealth interventions, there is growing evidence supporting the success of text-message–based programs (also known as SMS) in modifying health behaviors [6-9]. With >97% of Americans currently owning some type of cell phone and the prevalence of smartphone ownership having increased from 35% to 85% in the last 10 years [10], text messaging has become a staple mode of communication for most people in the modern world. Using a platform already embedded in most individuals’ routines, text messages eliminate the need for additional equipment or substantial behavior change. This universal reach and familiarity not only enhances patient engagement but also bridges the gap for underserved communities, thus playing a pivotal role in reducing health disparities [11-13]. Their omnipresent nature, readership and engagement advantage, and the ability to mirror the conversational tone of in-person counseling all underscore the unique value of text messaging in contemporary health interventions [14,15].

### Content Creation for Health Care

Content has been described as “the central driver of behavior change” in interventions [16], and its thoughtful incorporation through modalities like text [17,18], imagery [19,20], and other media [21,22], is key to effective intervention design. For brief message interventions in particular, textual content serves not only as a vessel for information, but also as the critical and emotional linchpin motivating behavior change. When we consider interventions designed to induce change, clarity in the content creation process becomes indispensable. It provides a coherent road map for both practitioners and researchers, ensuring that the outcomes of the intervention—successful or not—can be understood, dissected, and refined. Furthermore, a transparent process of content creation not only bolsters the effectiveness of an intervention but also builds trust within the broader scientific community, allowing for constructive critiques, replication of studies, and meaningful advancements in the field.

**Figure 1:**
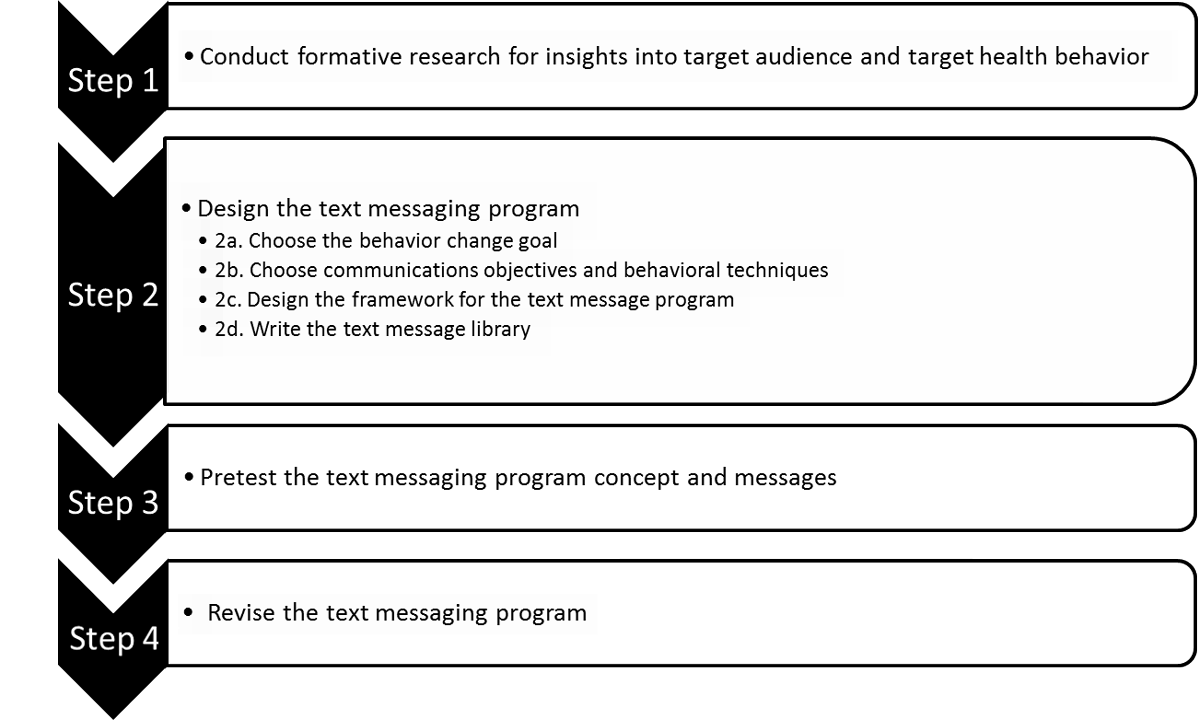
Text messaging program development pipeline (reproduced from from Abroms et al [23], which is published under Creative Commons Attribution 4.0 International License [24]).

The conventional SMS intervention development pipeline, shown in Figure 1, consists of the following parts: 1) formative research into the problem setting, behavior, and target population; 2) the establishment of the chosen theoretical framework and development of content; 3) a necessary review of the created content for quality assurance, safety, and research standards, as well as a pretest to gauge initial user feedback on the messages; and finally, a revision of messages based on accumulated feedback [23]. In this study, we address the second step of intervention design—the creation of content within a scientific and theoretical framework—due to its complexity and implication for specialists outside the traditional research team. Content designers can be used to greatly enhance the quality and efficacy of content for health care interventions; however, their involvement is often limited due to monetary, time, and resource constraints on the research team. Consequently, researchers are frequently tasked with taking on the roles of content designers themselves.

However, despite the involvement of the research team in content design and the critical importance of content, there exists a conspicuous opacity surrounding the creation of content in health care interventions. Numerous published works fall short in delineating the intricacies of their content creation processes while simultaneously withholding disclosure of their final message data sets, leaving a void in our understanding of how preliminary findings or formative research become translated into the finalized intervention—an omission that has led to the development process being described as a black box [25]. This lack of transparency is especially concerning given the tendency of some researchers to view text messaging as the intervention itself rather than just the means of delivery [26]. In those instances where the message creation process is disclosed, it often reveals a narrow involvement, typically limited to a few individuals within the intervention team [23,27]. Their varied levels of expertise in content design and differing perceptions of what constitutes “good content” can, as a result, lead to vast inconsistencies in outcomes that could be mistakenly attributed to other metrics like participant demographics, study duration, message volume, or the theoretical techniques used instead of the more crucial variable: the nature and quality of the content itself. In addition, this limited participation in content drafting tends to perpetuate familiar methodologies, sidelining innovative approaches that could potentially address persistent challenges like medication adherence [28]. Such exclusions not only hinder academic progress but could also inadvertently reduce the efficacy of interventions. When the foundation—content creation—is not soundly built with a clear and shared understanding of its underpinnings, it runs the risk of diluting the potential positive outcomes of the intervention. As brief message interventions continue to increase in number, complexity, and scope, the need for innovative and transparent approaches to content creation grows even greater.

### Generative AI With LLMs

Faced with the complexities and opacity of content creation, generative AI offers a promising solution to unveiling this enigmatic “black box.” Recent advancements in the development of LLMs using the transformer architecture [29] have brought about a revolutionary change in natural language processing. Unlike earlier models that process text sequentially, transformer models use a technique known as “self-attention” to analyze and draw connections between different parts of input data simultaneously. By converting text into corresponding numerical representations called embeddings, transformers can process language data with exceptional accuracy and speed. Furthermore, being pretrained on vast corpora of web-text data, these LLMs are not only equipped to simulate human conversations but also excel as versatile tools across a spectrum of nuanced tasks, such as question answering, writing support, translation, coding, and more [30-37].

Though the concept of data generation using LLMs is not novel in itself [38-40], the accessibility and enhanced generative capabilities of contemporary large-scale pretrained models like those in the GPT series have magnified their impact and broadened their potential applications [41,42]. With up to hundreds of billions of parameters [32,43], these models excel at rapidly generating vast quantities of contextually appropriate content, streamlining the traditionally painstaking process of manual drafting while simultaneously enhancing adaptability across diverse domains and sectors.

An integral aspect of effective LLM use lies in the art and science of prompt engineering. A prompt is any input given to an LLM that influences the nature of the LLM’s output [44]. Prompts are often given as sets of instructions or requests that establish the rules and guidelines of the conversation. Through prompt engineering, the context of the conversation can be strategically structured to direct the LLM to process relevant information and shape the desired form and content of its output [45]. This process is pivotal in refining and enhancing the capabilities of generative models and allows for the generation of more precise and relevant responses, which is especially imperative in complex fields like health care where the accuracy of information is essential.

Prompt engineering for LLMs is appropriate for the preliminary design of health care interventions for many reasons. First, LLMs can rapidly generate vast amounts of content, effectively reducing both the time and costs typically required for intervention development. This efficiency may allow researchers to allocate resources more appropriately, diverting their energies toward other critical aspects of the project while enabling the exploration of diverse content approaches that were previously considered daunting or impractical. Moreover, LLMs serve as a vital aid to researchers who may not have an extensive background in content design. By providing large amounts of well-written, contextually tailored content, these models offer a structured foundation that researchers can then build upon and further customize during the content review process while avoiding the overwhelm of “blank page paralysis” commonly inherent to creative tasks [46].

Perhaps most significantly for health care intervention research, the application of generative AI introduces a revolutionary level of transparency into the content creation process. By leveraging generative AI models as configurable tools, researchers gain access to a more standardized and reproducible approach for content design. This is primarily enabled through the adjustment of key parameters, such as the “temperature” setting, which are essential for tailoring the models’ outputs to specific needs [47,48]. A lower temperature results in more predictable and conservative outputs, while a higher temperature allows for increased variability and creativity in responses. Such configurability not only ensures reproducibility and accessibility but also allows for the establishment of standardized writing styles for health care interventions by minimizing the influence of tone, style, and other confounding variables. With a clearer understanding of the content generation process, researchers are better able to create content at scale, refine content with confidence, and make informed decisions that ultimately enhance the overall efficacy and impact of health care interventions.

### Medication Adherence for Type 2 Diabetes

We have chosen the setting of medication adherence for people with type 2 diabetes for our case study on the use of generative AI in health care interventions. Diabetes mellitus currently affects more than 415 million individuals worldwide, with an overwhelming 90% of these instances being attributed to type 2 diabetes [49,50]. Type 2 diabetes is often managed through a combination of dietary modifications, increased physical activity, and the consistent use of oral glucose-lowering medications. However, while oral antidiabetic medications are often critical to the management of type 2 diabetes, poor adherence to these medications is alarmingly common, with studies suggesting an average adherence rate of only 58% [51,52]. Recent attempts to address this issue have produced mixed results. Notably, a comprehensive review [53] of 182 randomized controlled trials focusing on interventions to improve medication adherence revealed that the evidence supporting their efficacy is largely unconvincing, despite many randomized controlled trials included in the review being extremely time- and resource-intensive. Consequently, such methods are challenging to scale and integrate into routine clinical settings. The paradoxical observation is that the increased complexity and costs of in-person, counseling-style intervention design might not directly lead to better adherence rates, resulting in a pressing need for more innovative, cost-effective, and scalable strategies.

In light of these concerns, SMS-based interventions have emerged as a promising avenue. These brief messaging interventions have previously demonstrated efficacy in promoting various health care behaviors [9,54-57]. Specifically in the domain of type 2 diabetes, interventions based exclusively on messaging [58-60] have shown encouraging results in enhancing medication adherence, though these findings are drawn from a limited number of trials and are not uniformly conclusive [61]. Furthermore, a limitation echoed in these studies is the notable absence of explicit theoretical frameworks guiding the interventions. For these SMS-based interventions to realize their full potential, it is paramount that they are founded on solid theoretical and technical bases, as adopting such an approach ensures that the behavioral mechanisms driving adherence are addressed effectively.

### Behavior Change Techniques

Described as the “active ingredients” of an intervention, behavior change techniques (BCTs) epitomize the most fundamental, replicable, and observable elements designed to modify the processes that regulate behavior [62,63]. To translate these strategies into a unified language, a taxonomy encompassing 93 BCTs organized into 16 groups was developed to guide behavior change interventions [64]. This standardization not only aids in replicating and optimizing strategies across various health behaviors but also enhances the comparability of research outcomes. By establishing which techniques are most effective under specific conditions, the taxonomy serves as a valuable resource for researchers and practitioners to select evidence-based approaches tailored to improving behavioral outcomes specific to their patient populations.

However, despite the taxonomy’s pivotal role in unifying terminology and subsequently facilitating more comprehensive correlations across behavior change interventions, the application of these BCTs in the realm of message-focused diabetes self-management research remains limited. Among the 93 BCTs outlined in the version 1 taxonomy, only a fraction has been used in published reports for this particular setting [65,66], despite evidence suggesting that interventions using more BCTs typically exert more substantial behavioral effects than those with fewer BCTs [67].

In light of this discrepancy, a comprehensive systematic review of systematic reviews was undertaken to quantitatively pinpoint various BCTs associated with medication adherence across chronic physical health conditions and qualitatively assess them in the context of type 2 diabetes [68]. Overall, the systematic review identified 46 BCTs pertinent to medication adherence in type 2 diabetes that can be used to develop direct messages for mobile devices to improve adherence among patients while simultaneously breaking down the various theoretical constructs (ie, variables from theories targeted by interventions) and mechanisms underlying specific behavioral strategies (ie, techniques not exclusively anchored to one theory but incorporated in interventions due to their predictive value in behavior). Therefore, from this systematic review, there emerges a robust theoretical foundation ripe for practical applications and explicitly suitable for crafting a bank of messages tailored for medication adherence among patients with type 2 diabetes.

## Methods

### Overview

This paper describes the use of generative AI to develop messages for patients with type 2 diabetes. When using generative AI for nuanced content creation tasks, understanding the context, requirements, and restrictions of the desired content becomes pivotal before initiating the development of a prompt.

### Context, Requirements, and Restrictions

#### Background

This section outlines the key theoretical and technical considerations of our study. Theoretically, we base our content on a preexisting systematic review and widely recognized content design standards to ensure appropriate selection of BCTs and address health disparities through standardized tone and readability. Technically, our focus is on the necessary constraints of SMS delivery systems and the use of a BCT database, which combines findings from the systematic review with fields from the BCT taxonomy to be used conjointly for prompt construction. The following subsections provide detailed insights into each of these aspects.

#### Problem Setting

Our setting is based on a rapid systematic review [68] identifying the theoretical constructs and behavioral strategies associated with medication adherence in people with type 2 diabetes and mapping them onto the BCT version 1 taxonomy [64]. The review was done in 2 stages: first, the quantitative review examined interventions and predictors of medication adherence, and second, the qualitative review focused on patients’ perceptions, beliefs, and decision-making related to medication adherence specifically for type 2 diabetes. Through this review, 20 theoretical constructs, 19 behavioral strategies, and 46 BCTs were identified as suitable for the content of brief messages to be delivered through mobile devices, which serves as a strong theoretical and scientific underpinning for determining the BCTs and communication objectives used in the content.

Note that the selection of elements used as a theoretical framework in this case study serves as a mere illustration of how one could transform the theoretical framework provided by the research team into a generative AI context. In other applications, the specific information at hand will differ, but the process of integrating such information into prompts may adhere to a comparable methodology.

#### SMS Standards and Limitations

**Figure 2:**
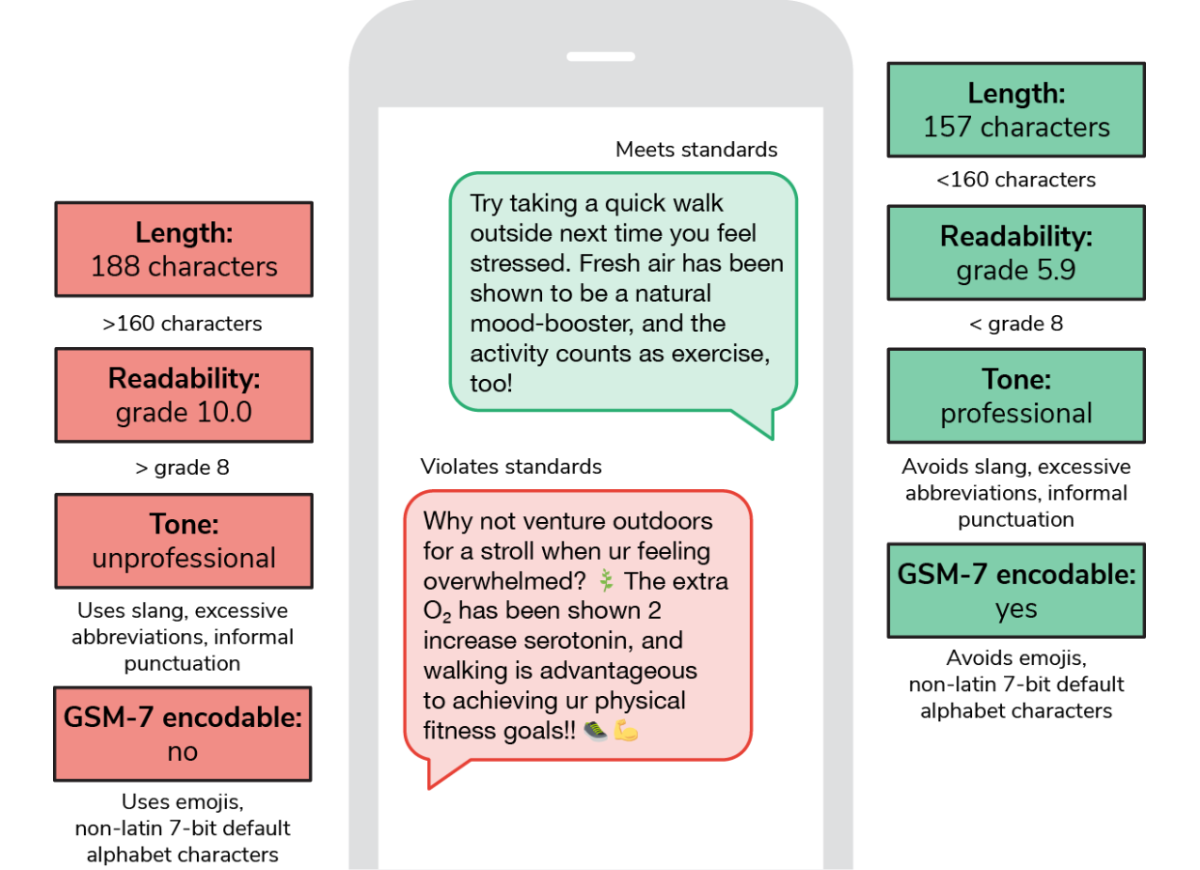
An example of messages violating (left) and meeting (right) SMS and content design standards. GSM-7: Global System for Mobile Communications.

Messages should ideally be 160 characters (including spaces) or less to be delivered as a single text message to a mobile phone and must consist of only Global System for Mobile Communications (GSM-7)–encodable characters (Figure 2). While some modern smartphones and mobile phone networks allow for message concatenation, enabling longer messages to be sent, requiring smartphone ownership for engagement in health care interventions has been shown to increase health disparities [69]. Thus, the restriction to the 160 characters encodable in GSM-7 has been used in this paper because it is the most standard restriction for SMS-based programs and allows for the greatest number of successful and predictable deliveries to participants.

#### Content Design Standards

As one must understand a message to be moved by it, literacy demands are a key focus in content design. Messages constructed using shorter words and sentences can cater to a wider range of literacy levels than those using advanced vocabularies and complicated sentence structures. While there are several metrics one might use to evaluate the complexity of a given text [70-73], to ensure accessibility and readability of the generated messages, they were assessed postcreation using the Flesch-Kincaid Grade Level Test due to its widespread use in practice and ease of implementation. The goal reading level is set within or below an 8th-grade level, which is considered the maximum recommended reading level for general adult audiences [74].

In addition, while text messages often carry an informal and conversational tone, in a health care context, even teenage audiences expect there to be a nuanced balance between the relaxed nature of the medium and the professional voice expected from a credible source [75]. Consequently, our messages are designed to avoid the use of slang, excessive abbreviations, or overly informal punctuation. At the same time, messages should convey warmth and friendliness, mirroring the knowledgeable tone of a health care professional with the approachability of a well-informed friend. Figure 2 shows a demonstration of appropriate and inappropriate content design.

#### BCT Database

To ensure consistency and replicability in the development of messages, we create a standardized database of the 46 BCTs selected based on the needs of the given setting as identified in the systematic review of brief message content [68]. The database contains comprehensive information on each BCT drawn from both the systematic review and the BCT Taxonomy version 1 [64]. By centralizing this data in one location, we can create uniform user prompts that are easily adjustable. This flexibility allows for structural modifications, the inclusion or omission of different fields, and swift adaptation if further curation of BCTs is required, thus ensuring both consistency and flexibility in the development of targeted health care interventions. There are six database fields:

1. Number—the number assigned to the BCT [64]
2. Label—the name of the BCT [64]
3. Definition—the definition of the BCT [64]
4. Examples—available examples of the BCT [64]
5. Theoretical constructs—theoretical constructs mapped to the BCT [68]
6. Behavioral strategies—behavioral strategies mapped to the BCT [68]

The final table containing the BCT database can be found in Multimedia Appendix 1.

### Technical Setup

To generate textual content, a pretrained LLM is needed. While a variety of options currently exist, both proprietary (eg, GPT [32] and LaMDA [76]) and open-source (eg, Orca [77] and Llama 2 [78]), we use GPT for this particular project. As one of the most advanced and widely recognized models in the field of AI-driven language generation [41,42,79,80], GPT benefits from an extensive body of research and a thriving community of developers.

In this work, we use the gpt-3.5-turbo-0301 model through OpenAI application programming interface (API) [47] calls to generate health care messages and use the chat completion API to communicate with the model. While the use of the completion functionality might appear to be more suitable for a single-prompt interaction, we observed superior results through the chat function during initial testing, and therefore continued development in a chat setting. Moreover, this choice aligns with the practical recommendations provided by OpenAI [48].

As our experiments are of an illustrative nature, we mostly use default parameter values for the API calls. In more nuanced use cases, these values could be tweaked by the prompt engineer to further tailor the output of the model to comply with the application, but adjustments were unnecessary for our use case. However, to ensure reproducibility of the presented results, we globally set temperature equal to 0, even though in practice one may obtain better outcomes by setting a positive temperature and rerunning the same query until a more satisfactory result is achieved. For instance, in ChatGPT, the value of the temperature is set to 0.7, which allows for more varied, human-like responses.

Message generation and analysis are performed in a Jupyter notebook using Python 3.8 on a consumer-grade laptop. The source code is included in Multimedia Appendix 1.

### Prompt Engineering

For this work, we consider single-prompt chat completion where the messages parameter contains 2 roles—“system” and “user”—and their corresponding “content.” The conversation begins with an initial system prompt, followed by a prompt from the user. The interaction concludes with a response from GPT, which provides the generated output. To enhance the performance of the model for a task with many constraints (in our case, these included length, complexity, style, and BCT incorporation), attributed prompt design [81] has been used for both the system and user roles.

The system role provides general context and behavior instructions to the assistant. It is used to explain the setting, rules, parameters, and personas of each participant in the conversation.

The content of our system prompt is given in Textbox 1 and consists of four main components:

1. Setting— this establishes the general setting of the conversation, that is, the designated roles of “user” (as behavioral scientist) and “assistant” (as diabetes specialist), and the goal of the interaction (to construct messages encouraging meditation adherence).
2. Style rules—these are guidelines on style to be used by the assistant when constructing messages. In this case, style rules focus mostly on the personality of the messages, in addition to limitations on length, complexity, and uniqueness.
3. BCT rules—these are guidelines on the incorporation of BCTs to be used by the assistant when constructing messages. BCT rules explain the importance of the BCT and give directions for use.
4. Task—this combines the previous 3 sections into a single, condensed statement defining the particular task being asked of the “assistant” role.

The user role begins the conversation by providing the first interaction to which the assistant role can respond. In our setting, the user role has been defined through the system prompt as “behavioral scientist,” and reflects a templatized version of the BCT database to provide the assistant with the selected BCT and its corresponding information.

The structure of our user prompt is given in Textbox 2, where the tokenized attributes are replaced with their corresponding values from the BCT database for each query. The five attributes used in our prompts are as follows:

1. bct_label: the name of the selected BCT [64], prepended by the label “BCT: ”
2. bct_definition: the definition of the selected BCT [64], formatted in line with the bct_label following an equal sign (=)
3. bct_examples: if available, examples of the selected BCT [64], formatted as a new line prepended by the phrase “For example, ”
4. bct_theoretical_constructs: if available, the theoretical constructs corresponding to the selected BCT [68], separated by 2 line breaks and prepended by the label “Theoretical Constructs: ”
5. bct_behavioral_strategies: if available, the behavioral strategies corresponding to the selected BCT [68], formatted as a new line and prepended by the label “Behavioral Strategies: ”

Results are delivered through the “assistant” role, which is the content generated by the chosen GPT model in response to each particular combination of system and user inputs. To maintain the consistency of the presented results, we postprocess the model output by stripping quotation marks and standardizing the message separation to a single line break.

**Attributed system prompt used for message generation.**

You are a Diabetes Specialist encouraging medication adherence in people with type 2 diabetes via brief messages.

Your messages are informed by different Behavior Change Techniques (BCTs).

I am a Behavioral Scientist who will describe the BCT you should use to frame your messages encouraging medication adherence.

Messages should be friendly and positive, but also professional, super short, and to-the-point. You are limited on space. Messages should be written at the reading level of an eighth grader. Word choice should be short and simple so everyone can understand. Every message must be entirely unique from all others in both language and structure.

The BCT I will provide is {bct_label}. It is the most important thing, and it is very nuanced. Messages must intelligently use {bct_label} to encourage medication adherence. All messages must prioritize {bct_label} over everything else. DO NOT write any part of the user message verbatim -- the BCT, theoretical constructs, and behavioral strategies are a secret.

Task: You will use these sets of rules to construct 25 diverse messages that use {bct_label} to increase medication adherence for recipients with type 2 diabetes.

**Attributed user prompt used for message generation.**

BCT: {bct_label} = {bct_definition}

For example, {bct_examples}

Theoretical Constructs: {bct_theoretical_constructs}

Behavioral Strategies: {bct_behavioral_strategies}

{bct_label} is critical to each creative, chatty message.

### Ethical Considerations

This research focused on the development of content for SMS interventions using generative AI, which did not require the collection or analysis of personal data or direct engagement with individuals. According to federal guidelines from the US Department of Health and Human Services, known as the “Common Rule” (45 CFR 46) [82], ethics board approval is required only for research on human subjects that entails obtaining data through interaction with individuals or the use of identifiable private information. Since this study did not meet these criteria, an ethics review was not applicable. The generated content and associated processes were evaluated for adherence to ethical standards in the context of AI-generated material and were designed to be transparent, reproducible, and free of harm.

## Results

### Overview

Using the attributed system and user prompts, 25 messages are generated for each of the 46 selected BCTs, resulting in 1150 BCT-informed messages. A total of 45,905 tokens costing US $0.07 were used for the entire data set generation, with an average of 998 tokens used and US $0.0015 expenditure per BCT. Approximately US $10 was spent for the entire prompt development process, which required over 300 iterations of the prompt. The first 5 generated messages for the first 6 BCTs are presented in Table 1, with the full data set made available in Multimedia Appendix 1.

We analyze the generated messages to evaluate their overall adherence to the required metrics. Specifically, we consider the following parameters: message length, message readability, and diversity of messages and their BCT alignments compared with an existing message data set.

**Table 1:** Examples of messages generated from the user query.

User query	Message generated	
1.2 Problem solving	Feeling overwhelmed by your medication regimen? Let’s work together to simplify it and make it more manageable. Are you experiencing any side effects from your medication? Let’s problem solve and find ways to cope with them. Let’s identify any barriers preventing you from taking your medication as prescribed and develop strategies to overcome them. Having trouble remembering to take your medication? Let’s problem solve and find ways to make it a habit. Are you concerned about the cost of your medication? Let’s problem solve and explore options for financial assistance.	
1.4 Action planning	Set a reminder on your phone to take your medication every day at the same time. This will help you remember to take it consistently. Think about the best time of day for you to take your medication and plan to take it at that time every day. Create a checklist of all the medications you need to take and when to take them. This will help you stay organized and on track. Plan to take your medication with a meal or snack to help prevent any stomach upset. Make a plan to refill your medication before you run out. This will ensure you always have it on hand.	
2.3 Self-monitoring of behavior	Keep track of your medication intake every day to ensure you are staying on track with your treatment plan. Make a habit of checking your blood sugar levels regularly to monitor the effectiveness of your medication. Keep a log of any symptoms you experience to help your health care provider adjust your medication as needed. Set reminders on your phone to take your medication at the same time every day to establish a routine. Use a medication tracker app to monitor your medication intake and set reminders for refills.	
2.4 Self-monitoring of outcome(s) of behavior	Keep track of your blood sugar levels every day to see how your medication is working for you. By monitoring your blood sugar levels, you can see the positive impact your medication is having on your health. Make a habit of recording your blood sugar levels to stay on top of your medication regimen. Keep a log of your blood sugar levels to help you stay motivated to take your medication as prescribed. By tracking your blood sugar levels, you can see how your medication is helping you manage your diabetes.	
2.7 Feedback on outcome(s) of behavior	Great job! By taking your medication as prescribed, you are helping to manage your diabetes and prevent complications. Keep up the good work! Taking your medication regularly can help lower your blood sugar levels and reduce your risk of long-term health problems. You are doing an amazing job! Remember, taking your medication as directed can help you feel better and stay healthy. Congratulations! By sticking to your medication regimen, you are taking control of your diabetes and improving your overall health. Way to go! Taking your medication on time and as prescribed can help you avoid complications and live a longer, healthier life.	

### Message Length

**Figure 3:**
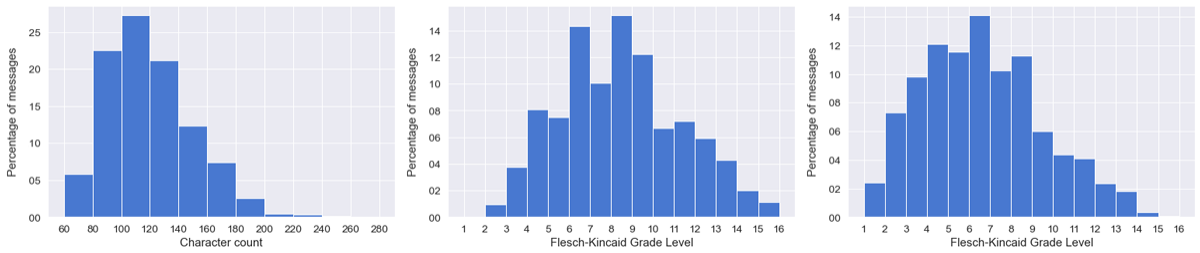
Length (left) and readability (before adjustment, center; after adjustment, right) of the generated messages.

Messages should be 160 characters or less. In total, 1034 of the 1150 (89.91%) total generated messages satisfy this requirement (Figure 3). The average length of all generated messages is 119 characters.

### Message Readability

Messages should fall within or below an 8th-grade level on the Flesch-Kinkaid Grade Level Test. In total, 688 of the 1150 (59.83%) total generated messages satisfy this requirement with no alterations for our setting (Figure 3). The average grade level of all generated messages is 8.4.

While this is the initial score for all messages, it is critical to note that the nonnegotiable and unsubstitutable word “medication” is considered complex due to its character length (10 characters) and number of syllables (4 syllables). However, for our setting, it is assumed that a population prescribed diabetes-management medications will be cognizant of the word “medication,” and it will thus not pose the same complexity barrier in our context as it might in other applications. Therefore, when the word “medication” is ignored during the readability calculation, 928 out of the 1150 (80.7%) total messages satisfy the readability requirement, with an average grade of 6.5 (Figure 3)—a closer, more accurate metric of complexity for our particular use case.

### Message Diversity

#### Overview

To evaluate the diversity of the generated messages, we compare them to the largest publicly available data set of SMS health care communications using BCTs to address behaviors surrounding diabetes [27]. We use pretrained natural language processing systems to compute the embeddings for each set of messages and compare their distribution.

It is important to note that due to the general opacity surrounding message creation for brief message interventions and the resulting lack of publicly available data sets, the study [27] we use for comparison is similar in theoretical framework used (BCTs) and general condition (diabetes), but different in population (individuals with prediabetes vs diagnosed diabetics), health behaviors addressed (diet and physical activity vs medication adherence), and size of the data set (124 vs 1150). Also note that some of the messages in the comparison data set [27] are coded for multiple BCTs. In such cases, we duplicate the message and assign each variation a single BCT to be consistent with our single-BCT-per-message mapping, resulting in a comparison data set consisting of 169 total messages.

#### BERT Embeddings and Principal Component Analysis Projection

We use BERT [83] to compute message embeddings through the bert-base-uncased model available through the Hugging Face Inference API [84]. For any message x∈X its BERT embedding vector emb(x) is given as emb(x)∈R^768^.

**Figure 4:**
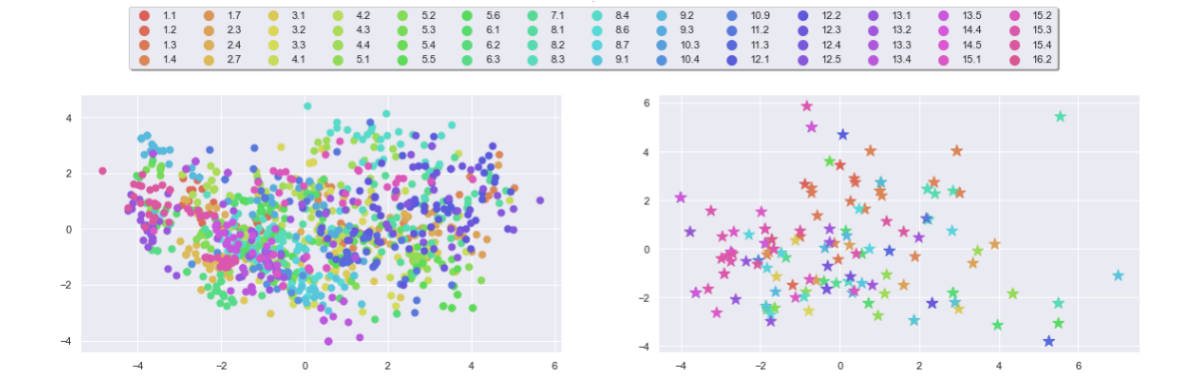
Principal component analysis projection of BERT embeddings of messages: ours (left) and comparison (right).

For each message, we compute its 768-dimensional embedding vector and then project it onto a 2D plane using principal component analysis (PCA) [85] (Figure 4). We note that the distributions of embeddings in both data sets are comparable, with embeddings being spread throughout the latent space without clustering per BCT, which indicates the presence of nontrivial semantic diversity.

#### ADA Embeddings and t-Distributed Stochastic Neighbor Embedding Visualization

**Figure 5:**
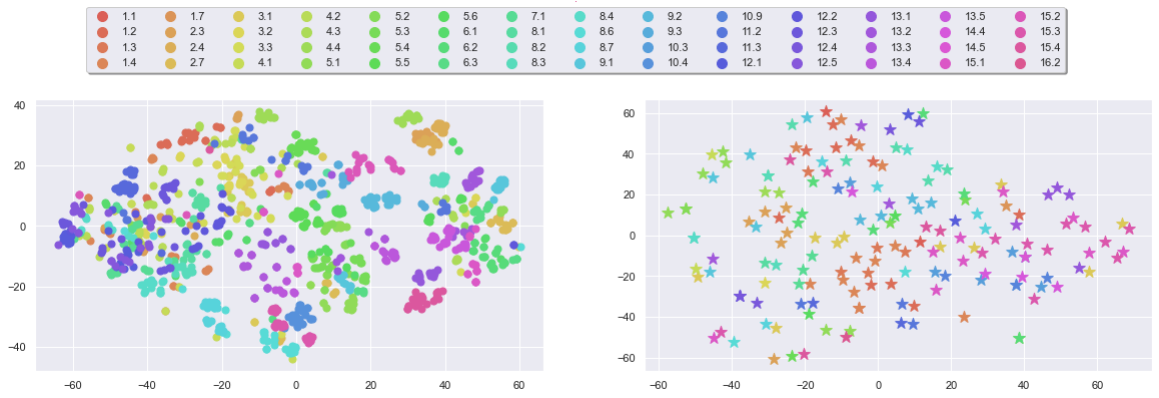
t-Distributed stochastic neighbor embedding visualization of ADA embeddings of messages: ours (left) and comparison (right).

We use ADA [86] to compute message embeddings through the text-embedding-ada-002 model available through the OpenAI Embeddings API [87]. For any message x∈X, its ADA embedding vector emb(x) is given as

emb(x)∈S^1535^⊂R^1536^,

where S^1535^ denotes the unit sphere in R^1536^. Because the embeddings computed by ADA are given as points on the unit sphere of the latent space, it does not seem sensible to use a linear projector like PCA; instead, we use t-distributed stochastic neighbor embedding (t-SNE) [88] to perform nonlinear dimensionality reduction. For each message, we compute its 1536-dimensional embedding vector and then embed them into a 2D plane using t-SNE (Figure 5). We note that the distributions of embeddings of both data sets are comparable, with messages corresponding to the same BCT being positioned closely.

#### Cross-Comparison of Data Sets

In this section, we aim to provide a more head-to-head comparison between the 2 data sets. To achieve such a comparison, 2 major differences in the data sets must be addressed: *distribution* (ie, the BCTs and number of messages per BCT) and *objective* (ie, the setting for which the messages are written).

To match the BCT-message *distribution* in 2 data sets, we first select the messages corresponding to the BCTs present in both data sets (Multimedia Appendix 1). Then, we check the number of messages available for each BCT in the comparison data set (169 messages mapped onto 41 BCTs) and take the same number of messages for each corresponding BCT from ours (1150 messages mapped onto 46 BCTs). This results in 2 sets of 135 messages spread across 31 BCTs (Multimedia Appendix 1).

While matching the objective is barely feasible, as it involves changing the semantic structure of each message, we attempt to nullify this difference by averaging the embeddings over each data set. Concretely, for each message x from the data set X we compute its representation *r*(x) by taking the ADA embedding emb(x) and centering it as,




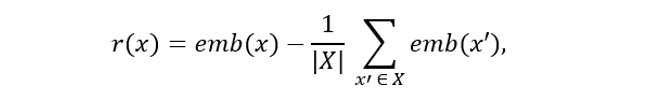

**(1)**



where X denotes the set of all messages from this data set and |X| denotes its cardinality. This modification is proposed in [89] and is prefaced on the assumption that the embedding emb(x) contains sufficient semantic information about the message x∈X, and thus the average of the embedding vectors over the data set represents the information that unifies all the messages, that is, the *objective*. By subtracting the average, the representation *r*(x) still contains the information that is specific to this particular message, that is, the semantic structure and the BCT.

**Figure 6:**
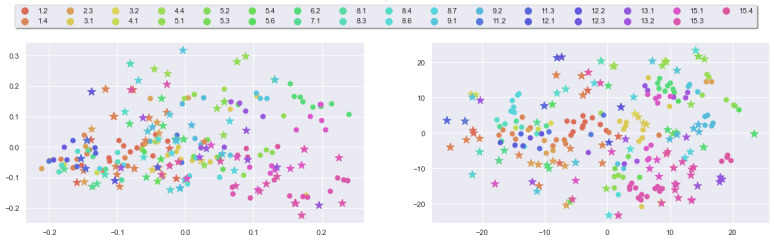
Projections of normalized ADA embeddings of messages: principal component analysis (PCA; left) and t-distributed stochastic neighbor embedding (t-SNE; right).

Computing representations (equation 1) for each message in both data sets allows us to directly compare the 2 data sets through the PCA and t-SNE projection of the message representations, shown in Figure 6.

Moreover, we compute the relevance between BCT encodings in the 2 data sets by averaging message representations corresponding to each BCT and then taking an inner product, that is,




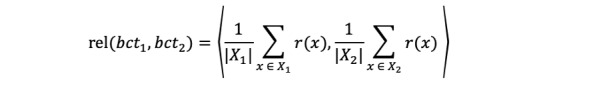

**(2)**



where ⟨·, ·⟩: R^1536^×R^1536^→R denotes the inner product, X_1_/X_2_ is the set of messages corresponding to BCT *bct*_1_/*bct*_2_, respectively, and *r*(x) is the numeric representation of the message x computed via equation 1. The resulting 31 × 31 heatmap can be found in Multimedia Appendix 1. The obtained relevancies can be used to evaluate the alignment of BCTs between 2 data sets, which result in a top-5 accuracy of 67% and a top-10 accuracy of 87%. Even though such an approach is a bit heuristic, we observe that the representations of the messages from both data sets are distributed similarly, often with messages corresponding to the same BCT being close to one another. This observation provides grounds to contend that the diversity of the messages generated by our approach is comparable to those previously created by researchers for practical, real-world applications.

## Discussion

### Overview

In this paper, we propose a novel approach to creating behaviorally informed content for brief message interventions. Using the setting of medication adherence for people with type 2 diabetes, we use a pretrained LLM to develop a bank of text messages based on BCTs curated in a recent systematic review [68]. This work is intended to act as a blueprint for future research to create a more transparent, replicable, and scientifically rigorous look into the content creation process for brief message interventions and serve as a starting point for subsequent studies to analyze the safety, efficacy, and viability of AI-generated messages.

### Principal Findings

In this paper, we show the potential of generative AI as a tool for transparent and replicable content creation in brief message interventions. Inspired by a list of 46 BCTs and their corresponding theoretical constructs and behavioral strategies, we engineer attributed system and user prompts for GPT to generate 25 messages for each of the 46 BCTs, for a total of 1150 messages in the specific setting of medication adherence for type 2 diabetes. Our findings reveal that a significant majority of the generated messages were compliant with both message length and complexity considerations (1034/1150, 89.91% and 928/1150, 80.7%, respectively), making them well-suited for SMS-style interventions.

The diversity of generated messages is analyzed through the distributions of their embedding vectors with 2 popular pretrained natural language processing systems: BERT and ADA. The generated messages showcase a diversity in message content that is comparable with an existing publicly available data set of brief messages and reflects similar distributions among BCTs from the comparison data set while also maintaining variability between messages of the same BCT, thereby demonstrating the capability for generative AI to craft a plethora of unique and contextually relevant communications with only a very standardized change in input between BCTs. The data set and source code for message generation and analysis are available in Multimedia Appendix 1.

### Technical Limitations

As generative AI is an extremely new field growing at a rapid rate, new algorithms and “versions” of LLMs are being released regularly. These updates can often fundamentally change the assistant output generated by the same system and user inputs, leading to a lack of consistency in experiments conducted with the same prompt over a long period of time. Especially for users of chatbot-style LLMs like ChatGPT, these updates can come suddenly and without permission, making research on such platforms difficult. While this issue is somewhat mitigated using an API (which generally does not force immediate adoption of the newest models), many current LLMs will eventually be depreciated, albeit at a more gradual rate. The only true mitigation of this limitation is the use of open-source models (such as, for example, Orca [77] and Llama 2 [78]) that can be fully downloaded and deployed on the client side; however, this comes at the cost of the technical proficiency required to set up such a system. Therefore, while prompts like ours serve as great examples of attributes to consider and the language one might use when constructing a prompt for a particular setting, the definitive construction of a singular, unchanging prompt to support intervention research is generally unfeasible in practice.

Another potential limitation of generative AI for very large-scale message generation is the finite context window size of the given LLM. In our results, we generate 25 messages per BCT, equating to an average of 998 tokens for each interaction, which keeps us well within the current 4000-token context limit bounds of gpt-3.5-turbo-0301. If a larger bank of messages is required, according to our current standardized single-prompt structure, one could feasibly increase the number of generated messages to 100 or more before nearing the limit. However, for extremely large-scale data generation, even a single-prompt interaction will likely be insufficient, and because LLM context is cumulative, this restriction will likely be most prominent for multistep interactions.

It is important to note that our use of a singular templatized prompt across multiple BCTs can sometimes fail to capture the nuanced essence of each distinct BCT. This “one-size-fits-all” approach may yield inconsistencies in the quality and accuracy of the generated content for certain BCTs as compared with others. However, prior studies in behavioral science have also suggested that some BCTs may not be capable of being delivered effectively in an SMS format regardless of the method of creation [90], perhaps indicating that difficulties in accurately representing some BCTs may have less to do with the limitations of generative AI and more to do with the inherent complexities of some BCTs in the given setting of brief message interventions.

Generative AI, while versatile, is heavily reliant on the specific language of the prompts provided; thus, when prompts rely heavily on external data fitting into a templatized structure, small, seemingly insignificant differences in the style or authorship of that data can potentially affect results [91,92]. For those aiming for AI-generated content that would be used in practice without human review, the design and fine-tuning of dedicated prompts for each unique contextual change (in our case, BCTs and their corresponding information) are likely a necessity. Therefore, while our approach serves as a proof of concept for efficient single-prompt content creation and demonstrates the vast potential of pretrained LLMs in this domain, achieving universally accurate results for all BCTs would necessitate a more granular, tailored approach to prompt design, attending to the individual nuances and requirements of each BCT within its specific context.

### Safety and Ethics in AI

As with any state-of-the-art technology, ethical considerations for the implementation of generative AI are paramount, guided by the core principles of transparency, privacy, accountability, and fairness [93,94]. However, the inherent unpredictability of LLMs becomes acutely significant in health care contexts, where the consequences of misinformation or inappropriate AI-generated suggestions can be dire for patients. Given that ensuring fully reliable, safe, and accurate information generation by LLMs is deemed “fundamentally impossible” [95], human checkpoints become indispensable before, during, and after AI employment. Researchers and domain experts must meticulously review AI-generated content for accuracy, safety, and equity, especially when evaluating its usability for complex patient-facing health care interventions.

It has been demonstrated that diversely attributed, complex prompts can reduce biases [81], but it is imperative to strike a balance, as excessively long prompts may increase the likelihood of undesired model behaviors [96]. In our approach, we acknowledge the crucial role of domain experts in both prompt design and content review, effectively mitigating the risk of malicious actor involvement and enabling the use of longer, more attributed prompts. Generative AI should not be seen as a standalone solution but as a tool that augments and accelerates the work of researchers. While it dramatically enhances efficiency in content creation, the responsibility for upholding rigorous standards of accuracy, safety, and fairness in health care interventions remains firmly with human experts, and it is the symbiotic collaboration between this tool and the human research team that ensures the delivery of ethically sound and clinically effective interventions.

### Comparison With Prior Work

To the best of our knowledge, this is the first work to propose the use of generative AI as a tool for content creation in SMS health interventions. However, a similar study [28] detailing a traditional content creation process was undertaken using the same systematic review [68] as a theoretical framework. A workshop was held for content creation and subsequent focus groups and surveys were used for review, resulting in the production of 371 messages informed by the selected BCTs in the context of medication adherence for type 2 diabetes. However, despite efforts toward transparency, this work does not reveal a detailed account of the actual content creation process, and the data set of generated messages has not been made publicly available for review or comparison.

Previous studies have looked at traditional content creation for brief message interventions, with a specific focus on the selection and review of BCTs and their corresponding messages [27,97,98]. More broadly, investigations into mHealth interventions have been a hot topic in health care research for years [11,14,15,26], and several works investigating the specific incorporation of behavioral science into brief message interventions have been previously undertaken with positive results [99-101].

In addition, one-shot, zero-shot, and few-shot approaches to prompt engineering have seen an explosion of interest following the expansion of LLMs within the public and academic mindset, leading to a large body of research on the methods and frameworks of prompt design for a variety of contexts and use cases [45,102,103].

### Future Work

This is the first in a series of works detailing the process for responsible and efficient use of generative AI in the development of brief message health care interventions. The next step involves assembling a team of qualified behavioral scientists and other domain experts to conduct in-depth analyses of the generated messages, focusing on their adherence to safety standards, adjustments to meet the technical requirements of an SMS delivery system, a formal review of BCT coding for each message, and general checks that the generated messages meet best practice standards for content design.

Moreover, the development of a subsequent interaction with the model could be used to self-adjust the generated results based on designer feedback. Using a multistep prompting method, the model could, for example, be directed to self-assess for safety and equity considerations, as well as edit more individually for the given use case based on specific critiques provided by the research team. Such iterative developments of the model should necessarily involve rigorous patient testing and feedback—a crucial step in ensuring that the AI-generated content resonates with patients’ experiences and needs to further personalize and refine the developed health care interventions.

Standardizing the realization of individual BCTs within brief message content represents another critical research direction. Many interventions currently withhold both their messages and their content creation processes, potentially introducing unintentional biases and skewed outcomes due to inherent differences in writing styles and other design-related confounding variables. By advocating for the transparency and standardization of content design, we can enhance the research efficacy of interventions by reducing such confounders and further ensuring that the results of such interventions are truly tied to the theoretical frameworks and behaviors being tested.

Finally, the ambitious goal of generating hyperpersonalized health care communications tailored to individual patients becomes a promising possibility with LLMs. Future iterations of content could be further personalized by implementing features like translating content for different languages and localizations; tailoring content for cultural relevance and sensitivity in the use of examples, metaphors, and references; adjusting the complexity levels of the text to cater to different educational backgrounds or cognitive abilities; and providing accessible formats adjusted for individuals with disabilities. While conventional content creation methods struggle with the impossible number of potential messages required for personalized content for every recipient, attributed prompting with generative AI offers the potential to create individualized messages that can significantly enhance patient engagement and outcomes and change the way health care interventions are experienced.

### Conclusions

In this study, we explore the practical application of generative AI for content creation in the development of brief message health care interventions. We illustrate the potential of using pretrained LLMs as a tool to aid researchers in the resource-intensive process of content creation by generating a data set of 1150 messages inspired by 46 BCTs selected for the setting of medication adherence for type 2 diabetes. Building on the foundations laid by former health care intervention development studies, this paper differentiates itself by the following:

1. Proposing and demonstrating the use of generative AI with pretrained LLMs for intervention development
2. Detailing the use of state-of-the-art AI tools for prompt engineering and content design processes
3. Providing the largest publicly available data set of messages created for SMS interventions, as well as the first publicly available source code offering fully transparent insight into the content creation process

Ultimately, the value proposition of using generative AI in this domain lies not in the perfection of the initial generated content but in its adaptability and capacity to rapidly produce a multitude of messages that can subsequently be refined and curated by human experts. This combination of AI-driven speed and human-driven supervision presents an efficient, transparent, and scalable method for developing effective and replicable brief message interventions. While follow-up studies are needed to ensure the safety and usability of the generated messages and provide potential refinements to the proposed prompts for individual settings, the use of generative AI in health care intervention development opens new doors for the scalability and potential standardization of content creation within health care intervention design and research. Given the time- and cost-intensive nature of crafting interventions traditionally and the current opacity of the content design process, our study underscores the potential of generative AI as a significant efficiency tool poised to revolutionize the creation of behavior change interventions for medication adherence and beyond.

## Appendix

Multimedia Appendix 1 Source code and data tables.

## Abbreviations

AI: artificial intelligence
API: application programming interface
BCT: behavior change technique
GSM-7: Global System for Mobile Communications
LLM: large language model
mHealth: mobile health
PCA: principal component analysis
t-SNE: t-distributed stochastic neighbor embedding
