# Supplementary figures and images for: Behavioral Nudging With Generative AI for Content Development in SMS Health Care Interventions: Case Study
Source: JMIR AI. 2024 Oct 15;3:e52974. doi: 10.2196/52974 (PMC11522651; doi:10.2196/52974)

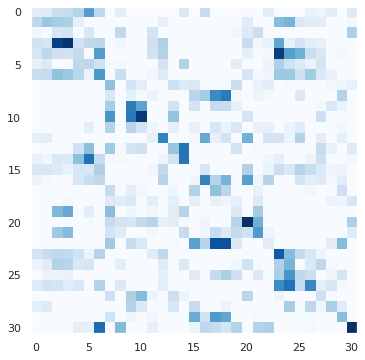

Supplement: Multimedia Appendix 1 [file ai_v3i1e52974_app1.zip › Multimedia Appendix 1/figures/embeddings_cross_heatmap.png]

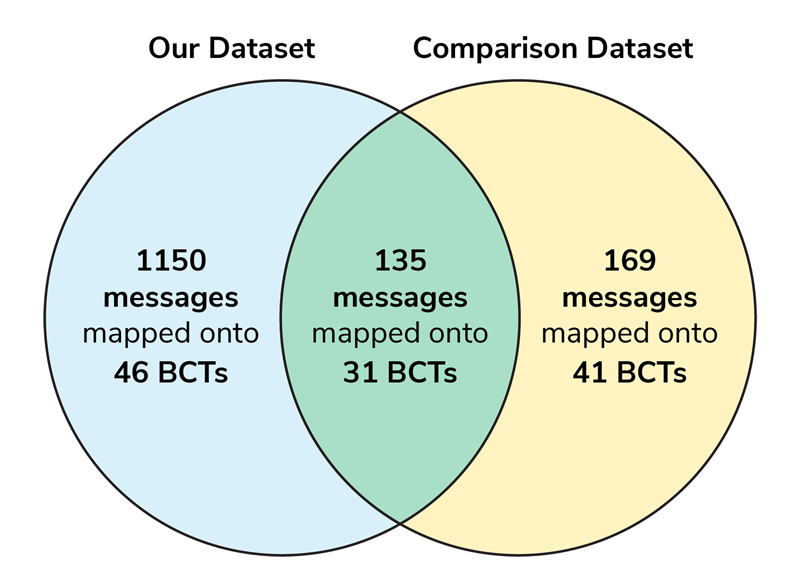

Supplement: Multimedia Appendix 1 [file ai_v3i1e52974_app1.zip › Multimedia Appendix 1/figures/dataset_size_comparison.png]
